# Life on a leaf

**DOI:** 10.7554/eLife.52123

**Published:** 2019-11-01

**Authors:** Robin Tecon

**Affiliations:** Department of Environmental Systems ScienceSwiss Federal Institute of Technology (ETH) ZürichZürichSwitzerland

**Keywords:** plant microbiome, bacterial survival, desiccation, *Pseudomonas fluorescens*, *Pseudomonas putida*, deliquescence, Other

## Abstract

Microscopic water films allow bacteria to survive the seemingly dry surface of plant leaves.

**Related research article** Grinberg M, Orevi T, Steinberg S, Kashtan N. 2019. Bacterial survival in microscopic surface wetness. *eLife* **8**:e48508. doi: 10.7554/eLife.48508

It may come as a surprise to many that plant leaves – from trees and crops to weeds and flowers – are heavily colonized by microorganisms, especially bacteria. Indeed, a gram of fresh plant leaf may harbor as many as a hundred million bacteria (Remus-Emsermann and Schlechter, 2018). This high number is even more surprising when you consider the extreme conditions that are found on the surface of a leaf – such as large changes in temperature, exposure to damaging UV light, and a shortage of nutrients and water. These conditions are particularly challenging during the day when leaves are dry. So, how do bacteria survive without water? One possibility is that some water remains, but only in the form of microscopic wetness that is invisible to the human eye. This wetness comes from water vapor that transpires from pores (also known as stomates) and condenses around particles on the surface of the leaf (Burkhardt and Hunsche, 2013).

Now, in eLife, Nadav Kashtan and colleagues at the Hebrew University of Jerusalem – including Maor Grinberg and Tomer Orevi as joint first authors and Shifra Steinberg – report how these thin water films increase the chances of bacteria surviving on plant leaves (Grinberg et al., 2019).

Observing bacteria directly on a leaf surface comes with a series of technical challenges. To avoid this, Grinberg et al. developed an experimental system that artificially mimicked the evaporation features of the leaf habitat. Surfaces covered with bacteria and thin liquid films could then be observed with microscopy using fluorescent dyes, which can distinguish between living and damaged or dead cells. Time-lapse microscopy enabled the researchers to see how micro-droplets dynamically formed around groups of bacterial cells.

To analyze the relationship between cell survival and water droplet size, Grinberg et al. studied a total of 13 species of bacteria, focusing on two species in particular, *Pseudomonas fluorescens* and *Pseudomonas putida*. Although there were quantitative differences between the species studied, all 13 species showed the same qualitative result: the chance of survival increased with the size of the water film (or droplet) surrounding the bacteria. Grinberg et al. suggest that this survival effect might result from the higher water potential (i.e. lower water stress) experienced by the bacteria in larger and thicker films.

Bacteria can live on plants leaves as solitary cells or in aggregates that contain up to thousands of cells. Previous experiments have shown that bacterial cells which are part of an aggregate are more likely to survive high levels of water stress (Monier and Lindow, 2003). In their study, Grinberg et al. hypothesized that the number and size of aggregates formed could itself have a role in retaining wetness on the surface of the leaf, and this may be why aggregation benefits survival. It was clear from their experiment that microscopic water droplets preferentially formed around aggregates and single bacteria, and that the size of the droplets positively correlated with the number of bacteria in aggregates. These results support the idea that the ability of aggregates to retain water contributes to cell survival.

The researchers then delved further into the physical mechanisms involved in microscopic surface wetness. This revealed that maintaining wetness relies on the presence of bacteria or other particles (such as resin microbeads) and also on the presence of solutes in the water droplets: in particular, microscopic wetness does not persist with pure water. This led Grinberg et al. to suggest that microscopic wetness is maintained by two simultaneous processes: surface tension that retains water around the particles, and solutes preventing the water from completely evaporating.

Of course, natural leaves are more complex than the artificial system used in this study, notably due to the microscale structures and chemical properties of their surfaces (Figure 1), so future research will need to explore more realistic surfaces (Doan and Leveau, 2015). However, with this study, Grinberg et al. have opened an exciting new window onto how bacteria are able to live on surfaces where moisture is limited. Far from being restricted to the leaf surface, habitats with low levels of water are common in both natural and built environments. The physicochemical processes investigated by Grinberg et al. therefore could help explain the ecological success of bacteria on surfaces beyond the plant leaf.

**Figure 1.**
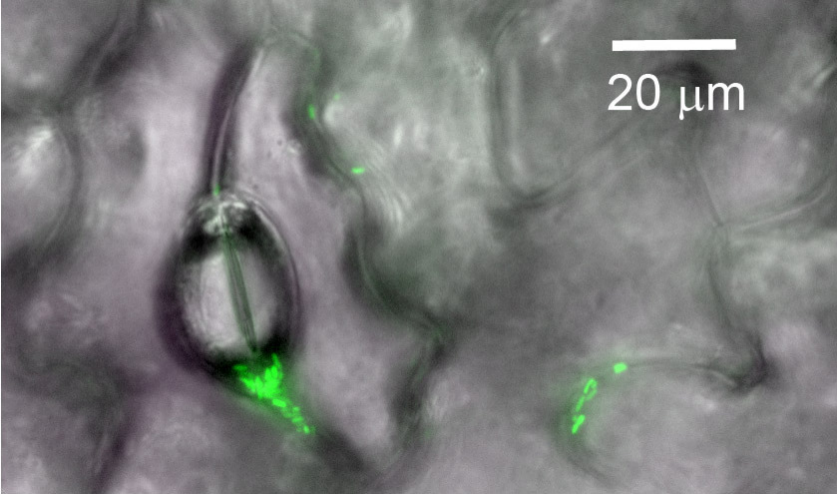
Bacterial cells on the surface of a plant leaf. Image showing single cells or aggregates of fluorescent bacteria (green) on the surface of a green bean leaf. Pores and grooves on the leaf surface are also visible.

Image credit: Robin Tecon.
